# Double cutoff strategies for plasma pTau217 to predict Tau PET positivity across multiple assay platforms: Tau-enriched and Tau-scarce cohorts for cost-effective clinical use

**DOI:** 10.1186/s13195-025-01952-y

**Published:** 2026-01-14

**Authors:** Heekyoung Kang, Yuna Gu, Jungah Lee, Soyeon Yoon, Henrik Zetterberg, Kaj Blennow, Fernando Gonzalez-Ortiz, Nicholas J. Ashton, Theresa A. Day, Michael W. Weiner, Daeun Shin, Sohyun Yim, Seonghyeon Kim, Hee Kyung Park, Min Young Chun, Eun Hye Lee, Jun Pyo Kim, Hee Jin Kim, Duk L. Na, Hyemin Jang, Sang Won Seo

**Affiliations:** 1 Department of Neurology, Samsung Medical Center, Sungkyunkwan University School of Medicine, 81 Irwon-ro, Gangnam-gu, Seoul, 06351 Republic of Korea; 2 Alzheimer’s Disease Convergence Research Center, Samsung Medical Center, 81 Irwon-ro, Gangnam-gu, Seoul, 06351 Republic of Korea; 3 Department of Psychiatry and Neurochemistry, Institute of Neuroscience and Physiology, the Sahlgrenska Academy at the University of Gothenburg, Medicinaregatan 3, 413 90, Göteborg, 40530 Sweden; 4 Clinical Neurochemistry Laboratory, Sahlgrenska University Hospital, Blå stråket 7, 413 45, Göteborg, 42180 Sweden; 5 Department of Neurodegenerative Disease, UCL Institute of Neurology, Queen Square, London, WC1N 3BG UK; 6 UK Dementia Research Institute at UCL, Queen Square, London, WC1N 3BG UK; 7 Hong Kong Center for Neurodegenerative Diseases, Clear Water Bay, Hong Kong, 53792 P.R. China; 8 Wisconsin Alzheimer’s Disease Research Center, University of Wisconsin School of Medicine and Public Health, University of Wisconsin-Madison, 600 Highland Ave, Madison, WI 53792 USA; 9 Paris Brain Institute, ICM, Pitié-Salpêtrière Hospital, Sorbonne University, 47 Boulevard de l‘Hôpital, Paris, 75013 France; 10 Neurodegenerative Disorder Research Center, Division of Life Sciences and Medicine, and Department of Neurology, Institute on Aging and Brain Disorders, University of Science and Technology of China and First Affiliated Hospital of USTC, 96 Jinzhai Road, Hefei, Anhui 230026 P.R. China; 11 Banner Alzheimer’s Institute and University of Arizona, Phoenix, AZ 85006 USA; 12 Banner Sun Health Research Institute, Sun City, AZ 85351 USA; 13 Centre for Age-Related Medicine, Stavanger University Hospital, Gerd-Ragna Bloch Thorsens gate 8, Stavanger, 4011 Norway; 14 Eli Lilly and Company, Lilly Corporate Center, Indianapolis, IN 46285 USA; 15 Department of Radiology and Biomedical Imaging, University of California, San Francisco, CA 94143 USA; 16 Department of Neurology, Yonsei University College of Medicine, 145-1, Jayang-ro, Gwangjin-gu, Seoul, 05025 Republic of Korea; 17 Department of Neurology, Yongin Severance Hospital, Yonsei University Health System, 225 Geumhak-ro, Cheoin-gu, Yongin-si, Gyeonggi-do 17046 Republic of Korea; 18 Department of Radiology and Imaging Sciences, Indiana University School of Medicine, 355 W 16th St, Indianapolis, IN 46202 USA; 19 Indiana Alzheimer Disease Research Center, Indiana University School of Medicine, 355 W 16th St, Indianapolis, IN 46202 USA; 20 Neuroscience Center, Samsung Medical Center, 81 Irwon-ro, Gangnam-gu, Seoul 06351 Republic of Korea; 21 Department of Digital Health, SAIHST, Sungkyunkwan University, 81 Irwon-ro, Gangnam-gu, Seoul, 06351 Republic of Korea; 22 Happymind Clinic, 23 Teheran-ro 87-gil, Gangnam-gu, Seoul, 06169 Republic of Korea; 23 Department of Neurology, Asan Medical Center, University of Ulsan College of Medicine, 88 Olympic ro 43 gil, Songpa gu, Seoul, 05505 Republic of Korea; 24 Department of Health Sciences and Technology, SAIHST, Sungkyunkwan University, 81 Irwon-ro, Gangnam-gu, Seoul, 06351 Republic of Korea; 25 Department of Intelligent Precision Healthcare Convergence, Sungkyunkwan University, 2066 Seobu-ro, Jangan-gu, Suwon, Gyeonggi-do 16419 Republic of Korea

**Keywords:** Plasma pTau217, Tau PET prediction, Double cutoff strategy, Diagnostic accuracy, DMT eligibility

## Abstract

**Background:**

We aimed to evaluate whether double cutoff strategies for plasma pTau217 across multiple assay platforms improve tau PET positivity prediction compared with single cutoffs and support cost-effective clinical use.

**Methods:**

We analyzed two cohorts: K-ROAD (*n* = 120; tau-enriched) and NA-ADNI (*n* = 280; tau-scarce). Tau PET positivity was defined within the temporal meta-ROI as SUVR > mean + 2 SD of Aβ-negative CU. Single and double cutoffs were compared for accuracy and misclassification-related costs.

**Results:**

Single cutoffs showed good accuracy across most assays (AUCs > 0.80), except for C2N ratio. Double cutoffs modestly improved accuracy but expanded intermediate groups, in the tau-scarce cohort. Cost analyses revealed assay- and cohort-specific effects, with reductions in most settings but increases for Lilly-MSD and Janssen-Simoa.

**Conclusion:**

Double cutoff strategies should be applied with attention to assay platform and cohort context. Accuracy gains were modest, but clinical utility lies in reducing misclassification costs and guiding confirmatory PET use.

**Supplementary Information:**

The online version contains supplementary material available at 10.1186/s13195-025-01952-y.

## Introduction

With the advent of disease-modifying therapies (DMTs) targeting amyloid-β (Aβ), such as anti-amyloid monoclonal antibodies, a growing number of patients are now receiving biologically tailored treatments in real-world settings [1–5]. These agents show greater efficacy in individuals with low or absent tau pathology [6–9], underscoring the need to accurately identify Aβ PET–positive individuals with low tau burden for optimized treatment selection. In parallel, tau-targeted therapies and combination strategies addressing both amyloid and tau pathologies are under development, further emphasizing the importance of precise tau stratification within the Aβ PET-positive population [4, 6–9].

Despite this need, routine tau positron emission tomography (PET) imaging remains costly and limited in accessibility, highlighting the necessity for scalable surrogate biomarkers [10–13]. Plasma phosphorylated tau 217 (pTau217) has emerged as a promising blood-based marker that reflects both amyloid and tau PET status [14–18], with prior studies, including ours, demonstrating its ability to delineate AT biomarker profiles across the Alzheimer’s disease (AD) continuum [14, 15, 19–21]. Recently, several clinical trials have adopted “biomarker-light” enrollment strategies that rely solely on blood-based markers such as pTau217, in lieu of PET imaging [19, 21–25]. Yet, despite its utility for detecting Aβ positivity, the ability of pTau217 to predict tau PET positivity using a single cutoff remains suboptimal, potentially limiting its clinical application in guiding treatment decisions [11, 14, 18, 19, 21].

Double cutoff strategies, which use 95% predictive blood test thresholds for Aβ positivity and negativity and define an intermediate zone between them, have been proposed to classify individuals into low, intermediate, and high plasma biomarker ranges [1, 14, 22, 26]. Compared to single cutoffs, the double cutoff strategy reduces misclassification of those with high and low values while ideally maintaining the intermediate group at a clinically acceptable level. This approach has been proposed to enable more reliable plasma-based prediction of tau PET positivity, particularly in heterogeneous clinical settings. Furthermore, various platforms for measuring pTau217 have been developed, each with differing analytic and predictive performance [13, 25, 27–31]. Whether the implementation of double cutoff models improves tau PET classification across these assays remains to be clarified.

In this study, we compared the performance of single versus double cutoff strategies for pTau217 in predicting tau PET positivity across two cohorts with differing tau burdens: the Korea–Registries to Overcome Alzheimer’s Disease and Accelerate Dementia (K-ROAD), a tau-enriched cohort (higher tau PET positive prevalence), and the North American Alzheimer’s Disease Neuroimaging Initiative (NA-ADNI), a tau-scarce cohort (lower prevalence) [14, 29]. Based on classification results, we also simulated therapeutic decision-making—such as assignment to amyloid-only or hypothetical combination therapy—and assessed the clinical and economic consequences of misclassification under each strategy [3, 10, 20].

## Materials and methods

### Study population

Participants were recruited from the Korea–Registries to Overcome dementia and Accelerate Dementia (K-ROAD) [32], a nationwide multicenter initiative conducted between 2016 and 2024. The K-ROAD study involves 25 tertiary-care hospitals across South Korea, with Samsung Medical Center serving as the coordinating center. Participants were recruited through both memory clinics and municipal dementia prevention centers to enhance the representativeness of the cohort. From the initially eligible K-ROAD participants with available tau PET scans, we excluded individuals diagnosed with non- Alzheimer’s clinical syndrome (*n* = 126) and those without plasma pTau217 data (*n* = 70). The final study cohort therefore included only individuals along the Alzheimer’s disease continuum—cognitively unimpaired (CU), mild cognitive impairment (MCI), and dementia. According to the 2024 NIA-AA revised criteria’s cognitive staging framework [33], updated by the 2024 NIA-AA revised criteria, participants were categorized into three groups: CU, MCI, and Alzheimer’s clinical syndrome – dementia stage. The CU group was defined by the following criteria: (1) absence of any medical history likely to impact cognitive function based on Christensen’s health screening criteria [6, 34], and (2) no objective cognitive impairment as measured by a comprehensive neuropsychological test battery across all cognitive domains (scores above − 1.0 standard deviation (SD) of age- and education-adjusted norms in memory and − 1.5 SD in other cognitive domains) [35]. Participants classified as MCI met the following criteria [32]: (1) subjective cognitive complaints from either the participant or their caregiver; (2) objective cognitive impairment in any domain (scores below − 1.0 SD of age and education-adjusted norms in memory and − 1.5 SD in other cognitive domains); (3) no significant difficulties in activities of daily living; and (4) absence of dementia. Participants classified as Alzheimer’s clinical syndrome – dementia stage met the clinical criteria for dementia as defined by the 2024 NIA–AA revised criteria, with a cognitive profile and clinical presentation consistent with Alzheimer’s disease but without biomarker confirmation [33]. All participants underwent [18 F]flortaucipir PET scans between May 2015 and December 2023. Additional assessments included standardized neuropsychological testing, structural MRI, and Aβ PET scans using either [18 F]florbetaben or [18 F]flutemetamol.

To assess the generalizability of our findings, we applied the same analytic framework to the NA-ADNI cohort, which had a lower tau positivity rate. This validation cohort comprised 280 participants who underwent similar neuropsychological assessments, [18 F]flortaucipir PET imaging, and MRI scans. Detailed inclusion and exclusion criteria for both cohorts are summarized in Supplementary Fig. 1.

We obtained written informed consent for the K-ROAD study and the institutional review board of each participating center approved the study protocol. Additionally, the ADNI Data sharing and publications committee approved data use and publication.

### Aβ PET acquisition and definition of Aβ PET positivity

All Korean participants underwent Aβ PET scans using either [18 F]florbetaben or [18 F]flutemetamol. PET imaging procedures were conducted in accordance with standardized protocols across participating K-ROAD centers. (Supplementary Method 1.1) All image analyses were performed at the central imaging laboratory of Samsung Medical Center.

Aβ uptake was measured using the Klunk Centiloid (CL) scale [34], which has been increasingly used in various cohort studies and clinical trials [6, 36]. Aβ PET positivity (hereafter referred to as Aβ-positive) was defined using a cutoff value of 20.0 for the study analysis. This threshold was selected to maintain methodological consistency with our previous work [29, 37] and is also supported by prior studies that have adopted 20 CL as a threshold for early amyloid positivity in preclinical and observational cohorts [38].

All NA-ADNI participants underwent [^18^F]florbetapir PET scans, and Standardized uptake value ratios (SUVRs) were obtained from the ADNI dataset (Supplementary Method 1.2). Previous studies [29, 36, 39] have used a cut-off of 1.11 SUVR for ADNI—calculated using the cerebellar cortex as the reference region—which converts to 19.8 CL using the equation CL = 188.22 × SUVR_florbetapir_ − 189.16 [29, 36].

### Tau PET data acquisition

[^18^F]Flortaucipir positron emission tomography/computed tomography (PET/CT) scans were conducted using two PET/CT systems: the Discovery STE PET/CT (GE Healthcare) at Samsung Medical Center and the Biograph mCT PET/CT (Siemens Medical Solutions) at Gangnam Severance Hospital. Participants received an intravenous bolus of approximately 280 MBq of flortaucipir, and PET imaging was initiated 80 min post-injection, lasting for 20 min. A head holder was employed to minimize motion artifacts during scanning, and a brain CT scan was acquired beforehand for attenuation correction. At Samsung Medical Center, PET images were reconstructed using an ordered-subset expectation maximization (OSEM) algorithm (6 iterations, 16 subsets) with a matrix size of 128 × 128 × 47 and voxel dimensions of 2.00 × 2.00 × 3.27 mm, whereas at Gangnam Severance Hospital, a matrix of 256 × 256 × 223 and a voxel size of 1.591 × 1.591 × 1 mm were used. For the ADNI cohort, all participants underwent tau PET using flortaucipir, with acquisition and processing protocols described in prior publications [40–42].

### Quantitative analysis of Tau PET and definition of Tau PET positivity

For both the K-ROAD and NA-ADNI cohorts, tau PET SUVRs were computed using FreeSurfer version 6.0 (http://surfer.nmr.mgh.harvard.edu/*)*, with the inferior cerebellar gray matter as the reference region. Partial volume correction (PVC) was not applied to ensure methodological consistency across cohorts and to avoid noise amplification and variability introduced by PVC algorithms [43, 44]. The primary region of interest (ROI) for defining tau PET positivity was the temporal meta-region of interest (temporal meta ROI), which included the entorhinal cortex, amygdala, fusiform gyrus, inferior temporal cortex, and middle temporal cortex. This ROI encompass early tau deposition areas such as the entorhinal cortex (Braak stages I–II) and adjacent temporal regions implicated in the progression of tau pathology.

Tau PET positivity (hereafter referred to as tau-positive) was defined within the temporal meta-ROI using a cutoff determined as the mean + 2 standard deviations (SD) of SUVR values from Aβ-negative cognitively unimpaired participants. The resulting thresholds were 1.339 in K-ROAD and 1.329 in NA-ADNI, showing minimal difference (0.01). To ensure comparability, we applied the NA-ADNI–derived cutoff of 1.329 uniformly across both cohorts [45–47]. Tau PET positivity was more prevalent in K-ROAD (61.7%) than in NA-ADNI (20.4%), supporting their designation as tau-enriched and tau-scarce populations, respectively. Although described as tau-enriched, the K-ROAD cohort included only two Aβ -negative, tau-positive participants, reflecting the study’s focus on tau PET positivity within the Alzheimer’s disease continuum. In contrast, NA-ADNI represents a predominantly low-tau prevalence setting; thus, analyses in this cohort primarily reflect classifier performance in a tau-scarce context, a key clinical scenario motivating the use of blood-based biomarkers as triage tools for confirmatory tau PET imaging.

### Plasma collection and processing

The detailed plasma collection and processing methods are described in Supplementary Method 2. Plasma pTau217 concentrations were measured using multiple assay–platform combinations across the two cohorts. In the K-ROAD cohort (*n* = 120), measurements were performed at the University of Gothenburg using the ALZpath antibody on the Simoa^®^ platform (UGOT-Simoa) and at Lilly Research Laboratories using a customized immunoassay on the Meso Scale Discovery platform (Lilly-MSD). After outlier exclusion (± 3 SD), all 120 participants had valid measurements for both assays. In the NA-ADNI cohort (*n* = 280), samples were analyzed at the Quanterix Accelerator Laboratory using the ALZpath antibody on the Simoa^®^ platform (Quanterix-Simoa), at C2N Diagnostics using the LC–MS/MS ratio of pTau217/non-pTau217 (C2N ratio), at Fujirebio using the Lumipulse^®^ G CLEIA platform with the proprietary pTau217 antibody (Fujirebio-Lumipulse), and at Janssen using a Simoa^®^-based assay with a Janssen-developed antibody (Janssen-Simoa). After excluding samples below the LOD (C2N: *n* = 106; Fujirebio: *n* = 7) and applying the ± 3 SD outlier criterion, the assay-specific analytic populations were as follows: Quanterix-Simoa (*n* = 280), C2N ratio (*n* = 171), Fujirebio-Lumipulse (*n* = 269), and Janssen-Simoa (*n* = 277). All measurements were performed in a blinded manner with standardized protocols, using a single batch of reagents per platform to minimize variability, and intra-assay coefficients of variation were < 10%.

### Cost-based evaluation of misclassification under different pTau217 cutoff strategies

We assessed misclassification of tau PET status using pTau217 cutoffs in Aβ–positive individuals, reflecting a treatment-allocation scenario in which anti-amyloid therapy—and potential add-on anti-tau therapy—would be considered only for Aβ-positive participants. For each assay and strategy, we calculated the number of false negatives (FN), false positives (FP), and individuals in the intermediate group. In the double cutoff model, the intermediate group was assumed to undergo confirmatory tau PET at a cost of 4,000 United States dollars (USD) per scan [10]. For FP cases, we assumed unnecessary administration of tau-targeted therapy, with a cost of 30,000 USD per person, based on the 18-month wholesale price of anti-amyloid monoclonal antibodies such as lecanemab [38], given that tau drug pricing is currently unavailable. For FN cases, we estimated an opportunity cost of 8,000 USD per person, assuming they would miss the clinical benefit of tau-targeted therapy. This was based on lecanemab’s estimated 0.08 quality-adjusted life years (QALY) gain and a 100,000 USD willingness-to-pay threshold [3, 48].

### Statistical analysis

We assessed the predictive performance of plasma pTau217 for tau -positive using receiver operating characteristic (ROC) curve analysis. For each assay, the area under the ROC curve (AUC) was calculated to quantify diagnostic accuracy. To classify tau PET positivity, we applied both single and double cutoff strategies separately in the K-ROAD and NA-ADNI cohorts. The single cutoff was determined using Youden’s index, maximizing the sum of sensitivity and specificity. For the double cutoff strategy, assay-specific procedures were used to define lower and upper thresholds in each cohort. In K-ROAD, cutoffs were first identified at 90% sensitivity (lower) and 90% specificity (upper), followed by refinement within ± 0.05 pg/mL intervals to increase robustness. Participants were then categorized into low, intermediate, and high groups. Performance metrics (accuracy, sensitivity, specificity, positive predictive value [PPV], and negative predictive value [NPV]) were calculated excluding the intermediate group. In NA-ADNI, refinement intervals were assay-dependent: ±0.5 units for C2N ratio, with additional ± 0.1 unit tuning; ±0.005 units for Janssen-Simoa; and ± 0.05 pg/mL for Quanterix-Simoa and Fujirebio-Lumipulse (consistent with K-ROAD).

All statistical analyses were conducted using SAS version 9.4 (SAS Institute Inc., Cary, NC). The data analysts were blinded to participants’ clinical diagnoses, Aβ PET status and tau PET status during evaluation.

## Results

### Baseline characteristics of the study population

Baseline characteristics are summarized in Table 1. The K-ROAD cohort (*N* = 120) had a mean age of 72.1 (Standard deviation [SD] 8.3), with 57.5% female and a mean education of 11.3 years (SD 4.5). Apolipoprotein E (*APOE*) ε4 carriers accounted for 54.2%, Aβ PET positivity was 83.3%, and tau PET positivity was 61.7%. The ADNI cohort (*N* = 280) had a mean age of 76.6 (SD 7.3), with 48.2% female and a mean education of 16.5 years (SD 2.7). *APOE* ε4 carrier rate was 33.2%, Aβ PET positivity was 48.6%, and tau PET positivity was 20.4%.

**Table 1:** Demographics and clinical characteristics of K-ROAD and NA-ADNI cohorts

Characteristics	K-ROAD (N = 120)	NA-ADNI (N = 280)
Age, years, mean (SD)	72.1 (8.3)	76.6 (7.3)
Female, n (%)	69 (57.5)	135 (48.2)
Education, years, mean (SD)	11.3 (4.5)	16.5 (2.7)
APOE ε4 carrier, n (%)	65 (54.2)	93 (33.2)
Clinical diagnosis, n (%)		
CU	26 (21.7)	172 (61.4)
MCI	41 (34.2)	81 (28.9)
Dementia	53 (44.2)	27 (9.6)
Aβ PET positive, n (%)	100 (83.3)	136 (48.6)
Clinical diagnosis among Aβ-positive participants, n (%)		
CU	14 (14.0)	73 (53.7)
MCI	34 (34.0)	39 (28.7)
Dementia	52 (52.0)	24 (17.6)
Tau PET positive, n (%)	74 (61.7)	57 (20.4)

*Abbreviations*: *K-ROAD *Korea–Registries to Overcome Alzheimer’s Disease and Accelerate Dementia, *NA-ADNI *North American Alzheimer’s Disease Neuroimaging Initiative, *N *Number, *SD *Standard deviation, *APOE4 *Apolipoprotein E ε4, *Aβ *Beta-amyloid, *CU *Cognitively unimpaired, *MCI *Mild cognitive impairment

### Single cutoff performance in predicting tau PET positivity

**Fig. 1:**
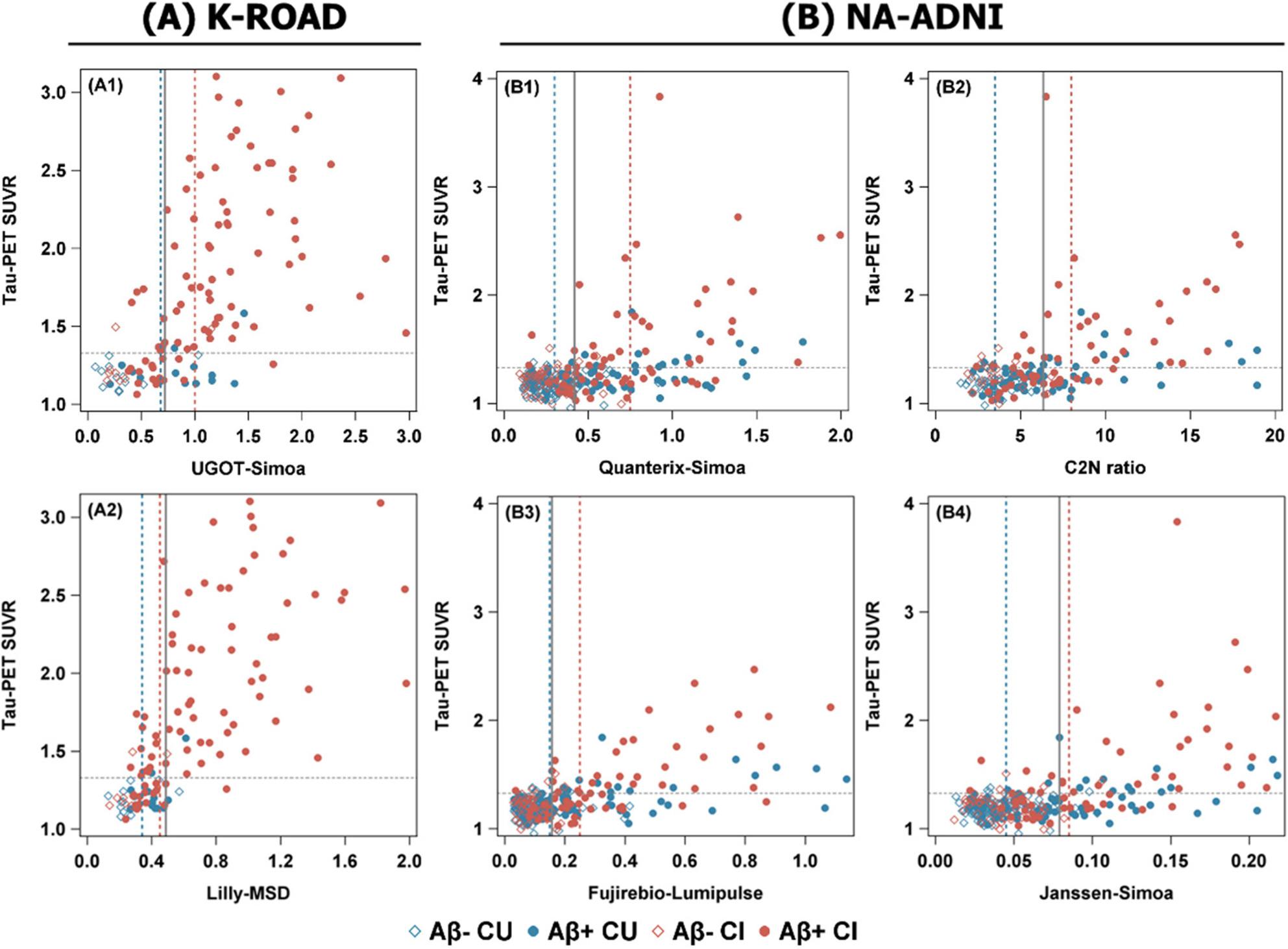
Scatter plots of plasma pTau217 versus tau PET SUVR across assays in two cohorts. Plasma pTau217 concentrations are plotted against tau PET SUVR. The vertical dashed lines indicate low and high cutoffs for cognitively unimpaired (CU) and cognitively impaired (GI) groups, respectively, while the solid vertical line denotes a single cutoff. The horizontal dashed line represents the tau PET positivity threshold (SUVR = 1.329). Results from the UGOT-Simoa and Lilly-MSD assays in the K-ROAD cohort (*N*=120) are shown in panels A1–A2, and results from the Quanterix-Simoa, C2N ratio, Fujirebio-Lumipulse, and Janssen-Simoa assays in the NA-ADNI cohort (*N*=280) are shown in panels B1 –B4. Abbreviations: pTau217, plasma phosphorylated tau 217; SUVR, standardized uptake value ratio; PET, positron emission tomography; CU, cognitively unimpaired; CI, cognitively impaired; K-ROAD, Korea–Registries to Overcome Alzheimer’s Disease and Accelerate Dementia; NA-ADNI, North American Alzheimer’s Disease Neuroimaging Initiative; UGOT-Simoa, ALZpath antibody on Simoa platform (University of Gothenburg); Lilly-MSD, customized pTau217 assay on MSD (Lilly Research Labs); Quanterix-Simoa, ALZpath antibody on Simoa platform (Quanterix); C2N ratio, LC–MS/MS–based pTau217/non-pTau217 ratio (C2N Diagnostics); Fujirebio-Lumipulse, pTau217 assay on Lumipulse G platform (Fujirebio); Janssen-Simoa, Janssen-developed pTau217 assay on Simoa platform (Janssen)

**Fig. 2:**
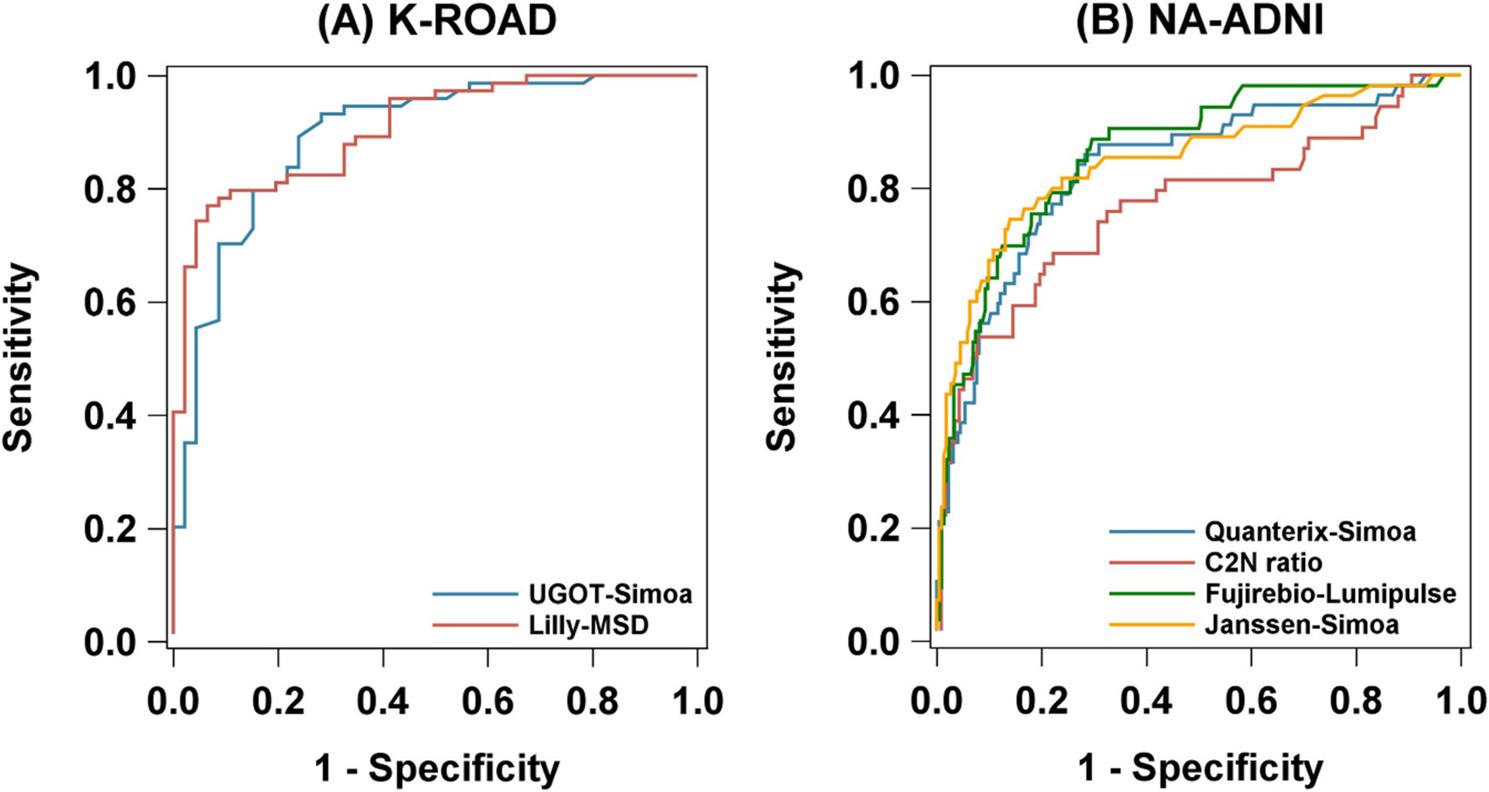
ROC curves of plasma pTau217 predicting tau PET positivity across assays in two cohorts. In the K-ROAD cohort (*N*=120) (A), the UGOT-Simoa assay showed an AUC of 0.833 and the Lilly-MSD assay an AUC of 0.907. In the NA-ADNI cohort (*N*=280) (B), the AUCs were 0.836 for Quanterix-Simoa, 0.767 for C2N ratio, 0.863 for Fujirebio-Lumipulse, and 0.849 for Janssen-Simoa, respectively. Abbreviations: AUC, area under the curve; pTau217, plasma phosphorylated tau 217; PET, positron emission tomography; K-ROAD, Korea–Registries to Overcome Alzheimer’s Disease and Accelerate Dementia; NA-ADNI, North American Alzheimer’s Disease Neuroimaging Initiative; UGOT-Simoa, ALZpath antibody on Simoa platform (University of Gothenburg); Lilly-MSD, customized pTau217 assay on MSD (Lilly Research Labs); Quanterix-Simoa, ALZpath antibody on Simoa platform (Quanterix); C2N ratio, LC–MS/MS–based pTau217/non-pTau217 ratio (C2N Diagnostics); Fujirebio-Lumipulse, pTau217 assay on Lumipulse G platform (Fujirebio); Janssen-Simoa, Janssen-developed pTau217 assay on Simoa platform (Janssen)

We evaluated single cutoff performance of plasma pTau217 in the K-ROAD (N = 120; UGOT-Simoa, Lilly-MSD) and NA-ADNI (N = 280; Quanterix-Simoa, C2N ratio, Fujirebio-Lumipulse, Janssen-Simoa) cohorts. As shown in Figure 1, the distributions of pTau217 levels by tau PET status are depicted, and optimal cutoffs derived from ROC analyses are indicated. The ROC analysis (Figure 2) demonstrated high concordance between pTau217 and tau PET, with AUCs of 0.833 (UGOT-Simoa) and 0.907 (Lilly-MSD) in K-ROAD, and 0.836 (Quanterix-Simoa), 0.767 (C2N ratio), 0.863 (Fujirebio-Lumipulse), and 0.849 (Janssen-Simoa) in NA-ADNI. At the optimal single cutoff, UGOT-Simoa (0.72) and Lilly-MSD (0.488) in the K-ROAD cohort showed sensitivity/specificity/PPV/NPV of 89.2/76.1/85.7/81.4 and 78.4/91.3/93.5/72.4, respectively. In the NA-ADNI cohort, Quanterix-Simoa (0.419), C2N ratio (6.36), Fujirebio-Lumipulse (0.157), and Janssen-Simoa (0.079) showed 86.0/71.7/43.8/95.2, 68.5/77.8/58.7/84.3, 88.7/70.4/42.3/96.2, and 74.5/86.0/56.9/93.2, respectively (Table 2).

**Table 2:** Comparative performance of plasma pTau217 assays using single and double cutoff strategies across cohorts

Cohort	Assay	Cutoff Type	Cutoff(s)	*N* total	T status (Low/High or Low/Intermediate/High)	Accuracy (%)	Sensitivity	Specificity	PPV	NPV
K-ROAD	UGOT-Simoa	Single	0.72	120	43 (35.8%)/77 (64.2%)	84.2	89.2	76.1	85.7	81.4
		Double	0.68/1.00	120	38 (31.7%)/24 (20.0%)/58 (48.3%)	88.5	91.2	84.6	89.7	86.8
	Lilly-MSD	Single	0.488	120	58(48.3%)/62(51.7%)	83.3	78.4	91.3	93.5	72.4
		Double	0.34/0.45	120	32 (26.7%)/23 (19.2%)/65 (54.2%)	88.7	81.8	90.8	90.8	84.4
NA-ADNI	Quanterix-Simoa	Single	0.419	280	168 (60.0%)/112 (40.0%)	74.6	86.0	71.7	43.8	95.2
		Double	0.30/0.75	280	118 (42.1%)/109 (38.9%)/53 (18.9%)	84.2	84.2	84.2	60.4	94.9
	C2N ratio	Single	6.36	171	108 (63.2%)/63 (36.8%)	74.9	68.5	77.8	58.7	84.3
		Double	3.5/8	171	47 (27.5%)/84 (49.1%)/40 (23.4%)	77	76.3	77.6	72.5	80.9
	Fujirebio Lumipulse	Single	0.157	269	158 (56.4%)/111 (39.6%)	74.0	88.7	70.4	42.3	96.2
		Double	0.15/0.25	269	154 (57.2%)/56 (20.8%)/59 (21.9%)	85.4	85.0	85.5	57.6	96.1
	Janssen-Simoa	Single	0.079	277	205 (73.2%)/72 (25.7%)	83.8	74.5	86.0	56.9	93.2
		Double	0.045/0.085	277	120 (43.3%)/93 (33.6%)/64 (23.1%)	82.6	86.4	81.4	59.4	95.0

*Abbreviations*: *K-ROAD *Korea–Registries to Overcome Alzheimer’s Disease and Accelerate Dementia, *NA-ADNI *North American Alzheimer’s Disease Neuroimaging Initiative, *UGOT-Simoa *ALZpath antibody on Simoa platform (University of Gothenburg), *Lilly-MSD *Customized pTau217 assay on MSD (Lilly Research Labs), *Quanterix-Simoa *ALZpath antibody on Simoa platform (Quanterix); C2N ratio, LC–MS/MS–based pTau217/non-pTau217 ratio (C2N Diagnostics); Fujirebio-Lumipulse, pTau217 assay on Lumipulse G platform (Fujirebio); Janssen-Simoa, Janssen-developed pTau217 assay on Simoa platform (Janssen); Cutoff, threshold value used to determine biomarker positivity; *N *Number, *T status *Tau PET status, *Accuracy *Classification accuracy; Sensitivity, true positive rate; Specificity, true negative rate, *PPV *positive predictive value; NPV, negative predictive value; pTau217, plasma phosphorylated tau 217; PET, positron emission tomography; Single, single cutoff strategy; Double, double cutoff strategy; Low/Intermediate/High, plasma pTau217 level classification based on cutoff values

### Double cutoff strategy for predicting tau PET positivity

**Fig. 3:**
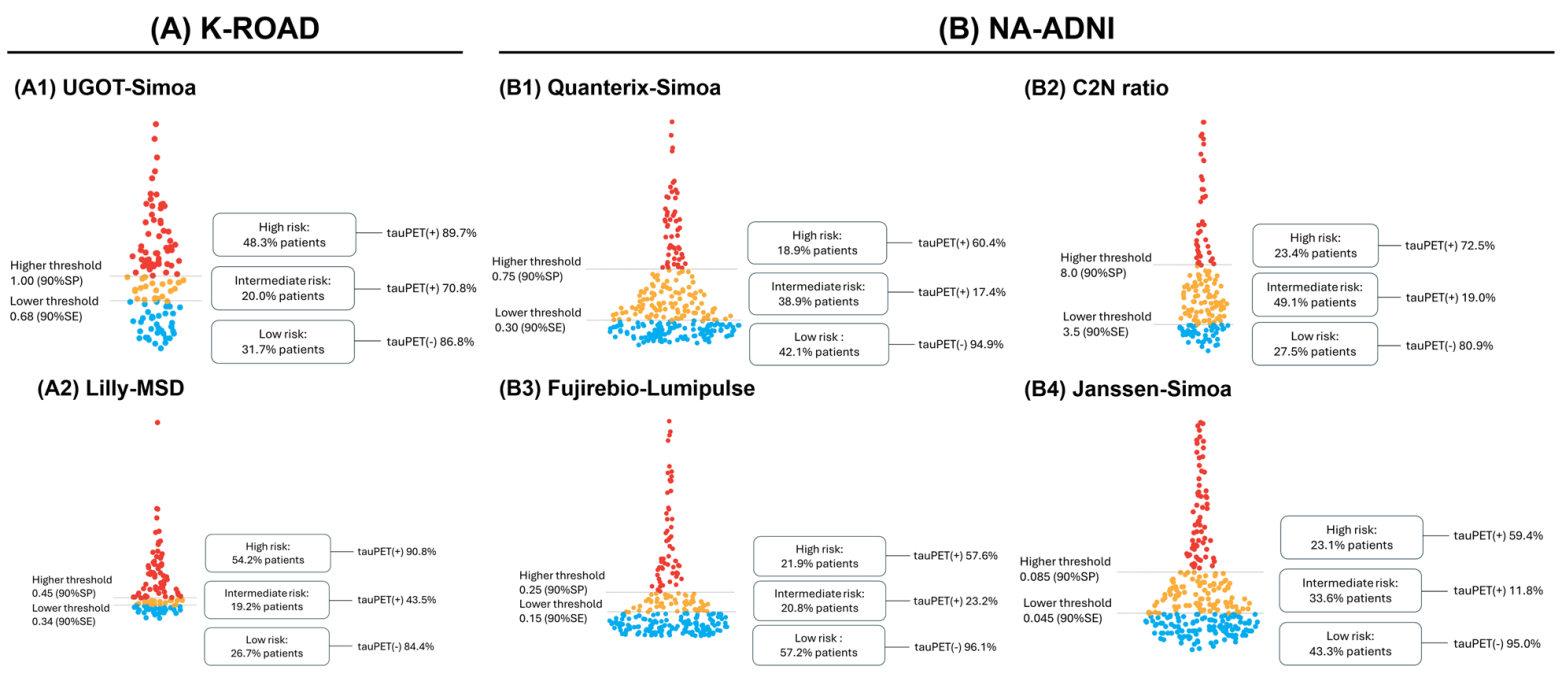
Distribution of plasma pTau217 by tau PET status and assay-specific double cutoffs in two cohorts. Plasma pTau217 levels across tau–positive (red) and tau–negative (blue) individuals are shown for each assay in the K-ROAD (A1–A2) and NA-ADNI (B1–B4) cohorts. Horizontal dashed lines indicate assay-specific double cutoffs, classifying participants into low, intermediate, and high groups. Abbreviations: pTau217, plasma phosphorylated tau 217; PET, positron emission tomography; K-ROAD, Korea–Registries to Overcome Alzheimer’s Disease and Accelerate Dementia; NA-ADNI, North American Alzheimer’s Disease Neuroimaging Initiative; UGOT-Simoa, ALZpath antibody on Simoa platform (University of Gothenburg); Lilly-MSD, customized pTau217 assay on MSD (Lilly Research Labs); Quanterix-Simoa, ALZpath antibody on Simoa platform (Quanterix); C2N ratio, LC–MS/MS–based pTau217/non-pTau217 ratio (C2N Diagnostics); Fujirebio-Lumipulse, pTau217 assay on Lumipulse G platform (Fujirebio); Janssen-Simoa, Janssen-developed pTau217 assay on Simoa platform (Janssen)

Using a double cutoff strategy that categorized participants into low, intermediate, and high groups, and reporting classification metrics as Accuracy/Sensitivity/Specificity/PPV/NPV (Figure 3, Table 2), UGOT-Simoa in the K-ROAD cohort classified 38 (31.7%)/24 (20.0%)/58 (48.3%) and showed 88.5/91.2/84.6/89.7/86.8, while Lilly-MSD classified 32 (26.7%)/23 (19.2%)/65 (54.2%) with 88.7/81.8/90.8/90.8/84.4. In the NA-ADNI cohort, Quanterix-Simoa classified 118 (42.1%)/109 (38.9%)/53 (18.9%) with 84.2/84.2/84.2/60.4/94.9; C2N ratio classified 47 (27.5%)/84 (49.1%)/40 (23.4%) with 77.0/76.3/77.6/72.5/80.9; Fujirebio-Lumipulse classified 154 (57.2%)/56 (20.8%)/59 (21.9%) with 85.4/85.0/85.5/57.6/96.1; and Janssen-Simoa classified 120 (43.3%)/93 (33.6%)/64 (23.1%) with 82.6/86.4/81.4/59.4/95.0.

### Cost-sensitive evaluation of single vs. double cutoff strategies

**Fig. 4:**
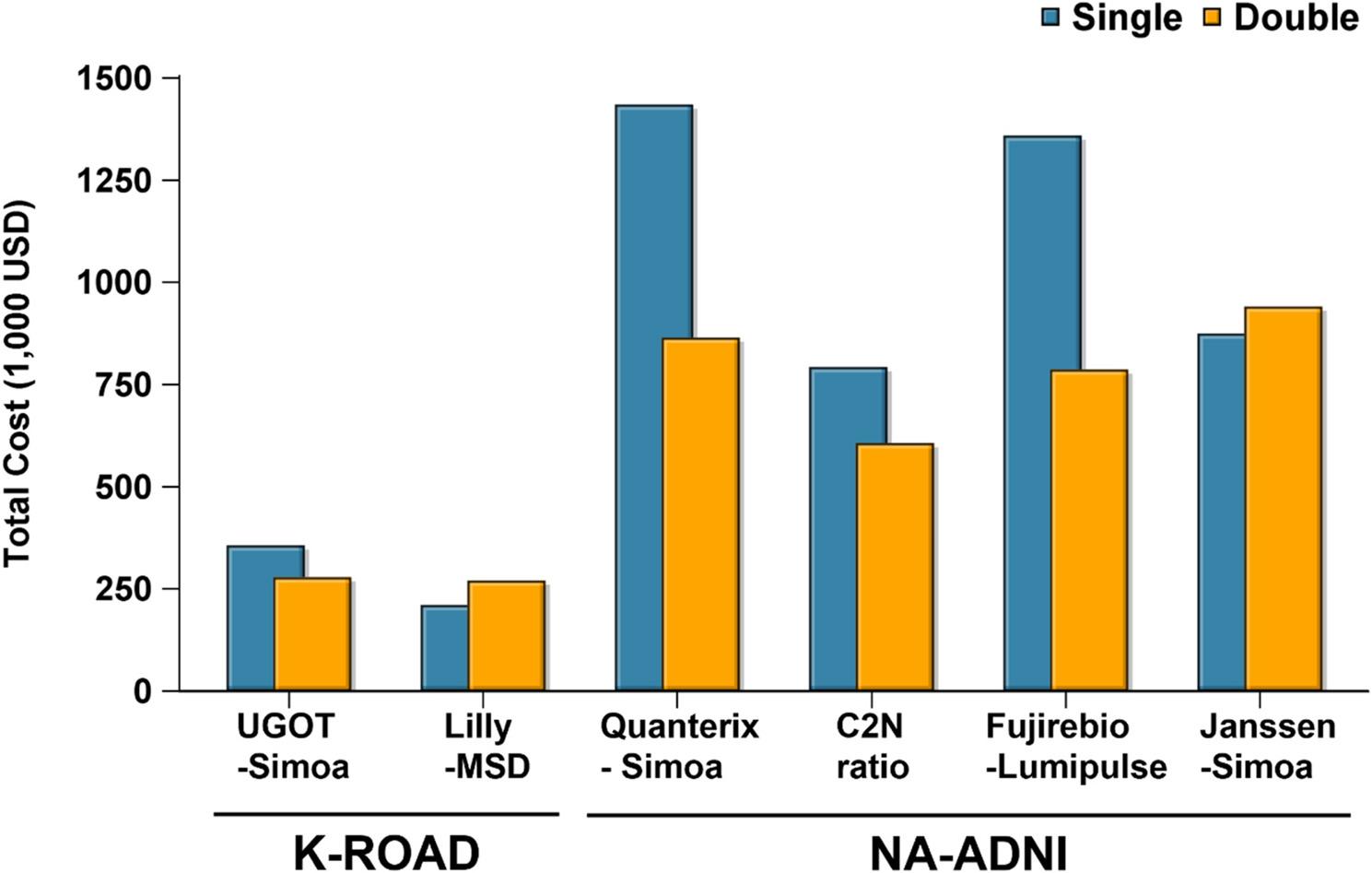
Total cost comparison of single and double cutoff strategies across pTau217 assays in Aβ-positive participants. Bar plots display the total cost (in 1,000 USD) associated with single (blue) and double (orange) cutoff strategies for each plasma pTau217 assay across two cohorts (K-ROAD and NA-ADNI). Costs were calculated based on the number of false negatives, false positives, and intermediate group using predetermined unit costs. All assays are based on pTau217 quantification. Abbreviations: UGOT-Simoa, ALZpath antibody on Simoa platform (University of Gothenburg); Lilly-MSD, customized pTau217 assay on MSD (Lilly Research Labs); Quanterix-Simoa, ALZpath antibody on Simoa platform (Quanterix); C2N ratio, LC–MS/MS–based pTau217/non-pTau217 ratio (C2N Diagnostics); Fujirebio-Lumipulse, pTau217 assay on Lumipulse G platform (Fujirebio); Janssen-Simoa, Janssen-developed pTau217 assay on Simoa platform (Janssen); K-ROAD, Korea–Registries to Overcome Alzheimer’s Disease and Accelerate Dementia; NA-ADNI, North American Alzheimer’s Disease Neuroimaging Initiative; pTau217, plasma phosphorylated tau

Table 3 and Figure 4 summarize the total costs associated with each strategy across plasma pTau217 assays in the K-ROAD and NA-ADNI cohorts. Importantly, these cost analyses were restricted to Aβ-positive individuals, as treatment allocation and misclassification costs are only clinically meaningful in this subgroup. In the K-ROAD cohort, UGOT-Simoa showed a reduction from 356 to 278 thousand USD (−21.9%), whereas Lilly-MSD exhibited an increase from 210 to 270 thousand USD (+28.6%). In the NA-ADNI cohort, Quanterix-Simoa, C2N ratio, and Fujirebio-Lumipulse demonstrated substantial cost savings, decreasing from 1,434 to 864 thousand USD (−39.7%), 792 to 606 thousand USD (−23.5%), and 1,358 to 786 thousand USD (−42.1%), respectively. By contrast, the Janssen-Simoa assay showed a modest increase from 874 to 940 thousand USD (+7.6%).

**Table 3:** Cost-based assessment of pTau217 cutoffs for therapy stratification in Aβ-positive participants

Cohort	Assay (*n*)	Strategy	FN (*n*)	FP (*n*)	Intermediate (*n*)	Total Cost (1,000 USD)
K-ROAD	UGOT-Simoa (*n* = 100)	Single	7	10	–	356
		Double	4	5	24	278
	Lilly-MSD (*n* = 100)	Single	15	3	–	210
		Double	4	5	22	270
NA-ADNI	Quanterix-Simoa (*n* = 136)	Single	3	47	–	1,434
		Double	2	20	62	864
	C2N ratio (*n* = 114)	Single	9	24	–	792
		Double	4	11	61	606
	Fujirebio-Lumipulse (*n* = 132)	Single	1	45	–	1,358
		Double	1	21	37	786
	Janssen-Simoa (*n* = 133)	Single	8	27	–	874
		Double	2	24	51	940

*Abbreviations*: *pTau217 *plasma phosphorylated tau 217, *K-ROAD *Korea–Registries to Overcome Alzheimer’s Disease and Accelerate Dementia, *NA-ADNI *North American Alzheimer’s Disease Neuroimaging Initiative, *UGOT-Simoa *ALZpath antibody on Simoa platform (University of Gothenburg), *Lilly-MSD *Customized pTau217 assay on MSD (Lilly Research Labs), *Quanterix-Simoa *ALZpath antibody on Simoa platform (Quanterix). *C2N ratio *LC–MS/MS–based pTau217/non-pTau217 ratio (C2N Diagnostics), *Fujirebio-Lumipulse *pTau217 assay on Lumipulse G platform (Fujirebio); Janssen-Simoa, Janssen-developed pTau217 assay on Simoa platform (Janssen), *FN *False negative, *FP *False positive, *USD *United States dollar

As sensitivity analyses, we additionally performed cost-based evaluations including both Aβ-positive and Aβ-negative participants to account for the full biomarker spectrum, including available Aβ-negative, tau-positive profiles (Supplementary Table 1). As expected, absolute cost estimates differed from the primary analyses restricted to Aβ-positive individuals; however, the relative assay- and cohort-specific cost patterns were largely preserved. When analyses were further restricted to Aβ-positive CI participants, similar cost advantages of double cutoff strategies were observed in the tau-scarce NA-ADNI cohort—except for Janssen-Simoa—whereas results in the tau-enriched K-ROAD cohort showed greater assay-specific variability (Supplementary Table 2).

## Discussion

In this study, we compared single versus double cutoff strategies for plasma pTau217 across multiple assay platforms in two cohorts with distinct tau PET prevalence—one tau-enriched (K-ROAD) and one tau-scarce (NA-ADNI). Our major findings are as follows. First, using a single cutoff, plasma pTau217 demonstrated good accuracy in predicting tau PET positivity across most assay platforms (AUCs >0.80), except for the C2N ratio. Second, double cutoff strategies improved accuracy across most assays compared to single cutoffs, though they did not fully meet the confirmatory testing standards proposed by the CEO initiative. Finally, among Aβ-positive individuals, cost analyses showed assay- and cohort-specific effects, with generally greater savings in the tau-scarce cohort but increases for Lilly-MSD in the tau-enriched cohort and Janssen-Simoa in the tau-scarce cohort. Taken together, our findings suggest that double cutoff strategies predicting tau PET positivity should be applied with careful attention to both cohort type and assay platform. While accuracy gains were modest, their greatest clinical value may lie in reducing misclassification-related costs—particularly by minimizing unnecessary confirmatory PET scans in resource-limited or tau-scarce settings. These results emphasize the combined importance of assay-specific approaches and cohort context, and highlight the need for more tau-specific blood biomarkers to refine future clinical applications.

Our first major finding was that plasma pTau217, when using a single cutoff, demonstrated good accuracy in predicting tau PET positivity across most assay platforms (AUCs >0.80), with the exception of the C2N ratio (AUC = 0.767). This finding is consistent with previous work, including our recent study [20], which showed that plasma pTau217 effectively delineates AT biomarker profiles across the AD continuum. The strong performance of pTau217 likely reflects its close association with AD-specific tau pathology [49–52]. Compared to several other phosphorylated tau species, pTau217 has shown the strongest correlation with brain tau accumulation [4, 13, 23, 28, 49, 50, 53], potentially due to its linkage with tau phosphorylation events occurring early in the disease cascade. This is consistent with the biological hypothesis that abnormal phosphorylation and release of tau proteins during neurodegeneration contribute to elevated plasma levels, thereby allowing pTau217 to serve as a peripheral marker of brain tau burden [17, 52].

Our second major finding was that double cutoff strategies—defining low, intermediate, and high plasma pTau217 ranges—improved accuracy across most assay platforms compared to single cutoffs; however, they did not fully meet the confirmatory testing standards proposed by the CEO initiative (i.e., sensitivity and specificity >90%, intermediate group <20%). Notably, while the intermediate group remained manageable (~20%) in the tau-enriched K-ROAD cohort, it expanded substantially in the tau-scarce NA-ADNI cohort, ranging from 36.8% to 49.1% except for Fujirebio-Lumipulse (20.8%). This pattern is unlikely to be explained solely by assay differences, as both cohorts included assays with the same antibody–platform combination. Specifically, the ALZpath antibody was measured using the Simoa platform in both cohorts (UGOT-Simoa in K-ROAD and Quanterix-Simoa in NA-ADNI). Instead, this discrepancy highlights the critical role of cohort context—particularly the prevalence and distribution of tau pathology—in shaping the discriminative performance of plasma pTau217, with greater overlap between tau-positive and -negative individuals in low-tau settings leading to increased diagnostic ambiguity.

Our final major finding was that, among Aβ-positive individuals, the impact of double cutoff strategies on cost varied substantially across assay platforms. In the tau-enriched K-ROAD cohort, double cutoffs modestly reduced costs with UGOT-Simoa but increased costs with Lilly-MSD, underscoring platform-specific differences. By contrast, in the tau-scarce NA-ADNI cohort, double cutoff strategies resulted in meaningful reductions in misclassification-related costs, with the greatest savings observed for Fujirebio-Lumipulse and Quanterix-Simoa, and more modest reductions with the C2N ratio. However, Janssen-Simoa did not yield cost savings despite improved accuracy. These assay- and cohort-specific cost patterns were largely preserved in sensitivity analyses that included the full biomarker spectrum or were restricted to Aβ-positive CI participants, reinforcing the robustness of the observed advantages of double cutoff strategies in tau-scarce settings. Importantly, their greatest clinical value may lie in reducing misclassification-related costs—particularly by minimizing unnecessary confirmatory PET scans in resource-limited or tau-scarce settings. These findings suggest that incorporating double cutoffs into clinical decision-making frameworks may enhance cost-effectiveness by minimizing inappropriate therapy allocation, especially as combination therapies targeting amyloid and tau are increasingly investigated. While tau-targeted therapies are not yet clinically approved, such strategies are actively being investigated, and precise assessment of tau status will likely become crucial for optimal therapeutic planning [2, 8, 30, 51]. In this context, blood-based triaging with double cutoff models could provide a scalable, efficient approach to guide confirmatory tau PET imaging, improving treatment targeting while reducing healthcare expenditures.

A key strength of this study lies in its comprehensive evaluation of both single and double cutoff strategies across two independent cohorts with differing tau burdens, using multiple plasma pTau217 assay platforms. However, several limitations should be noted. First, although the K-ROAD and NA-ADNI cohorts were intentionally selected to represent tau-enriched and tau-scarce populations, respectively, several factors may limit the generalizability of our findings. Overlap in assay platforms between cohorts was limited—although ALZpath on the Simoa platform was available in both—restricting direct platform-level comparisons across different tau prevalence settings. In addition, marked differences between the two cohorts in demographics, APOE ε4 frequency, and Aβ and tau PET prevalence likely contributed to the observed variability in cutoff performance. These findings indicate that plasma pTau217 thresholds are inherently dependent on the underlying biological context of the target population and should not be applied as universal cutoffs. Consequently, optimal cutoff calibration and performance may differ in less selected and more heterogeneous settings, such as primary care or community-based populations, where comorbidities, non–Alzheimer’s disease pathologies, and lower pretest probabilities are more prevalent. This limitation primarily affects generalizability rather than internal validity and underscores the need for cohort-specific calibration and external validation in broader real-world populations [12, 18, 54]. Second, although we examined economic implications, our cost estimates were model-based and illustrative rather than definitive, as they relied on hypothetical assumptions regarding future tau-targeted therapies and imaging pathways. Accordingly, this analysis was intended to compare the relative impact of misclassification between single and double cutoff strategies under a unified framework, with plasma pTau217 positioned as a triage tool to optimize confirmatory tau PET use rather than to replace molecular imaging. Finally, our findings should therefore be interpreted as reflecting tau PET prediction performance primarily within the AD continuum, rather than as evidence of specificity for AD-related tau pathology across all biomarker profiles.

In summary, double cutoff strategies for plasma pTau217 optimize performance by cohort type—enhancing accuracy in tau-enriched settings and cost-efficiency in tau-scarce settings—while maintaining a stable proportion of indeterminate cases. These tailored benefits suggest that double cutoffs can better align biomarker interpretation with underlying disease burden. Such an approach may guide more practical and resource-conscious use of tau PET in clinical and research contexts.

## Supplementary Information

Supplementary Material 1.

## Data Availability

The datasets used and/or analyzed during the current study are available from the corresponding author on reasonable request.
