# Supplementary material for: Double cutoff strategies for plasma pTau217 to predict Tau PET positivity across multiple assay platforms: Tau-enriched and Tau-scarce cohorts for cost-effective clinical use
Source: Alzheimers Res Ther. 2026 Jan 14;18:33. doi: 10.1186/s13195-025-01952-y (PMC12888305; doi:10.1186/s13195-025-01952-y)
Supplement: Supplementary file 1 — Supplementary Material 1. [file 13195_2025_1952_MOESM1_ESM.docx]

**SUPPLEMENTARY MATERIALS**

[Supplementary Methods 2](#_Toc214817456)

[**1. Protocols of Aβ PET imaging acquisition** 2](#_Toc214817457)

**2. Protocols of plasma collection for the K-ROAD study** 4

[Supplementary Tables 5](#_Toc214817459)

[**Supplementary Table 1. Cost-based assessment of pTau217 cutoffs for therapy stratification including Aβ-positive and Aβ-negative participants** 5](#_Toc214817460)

[**Supplementary Table 2. Cost-based assessment of pTau217 cutoffs for therapy stratification in Aβ-positive cognitively impaired participants** 7](#_Toc214817461)

[Supplementary Figure 9](#_Toc214817459)

[**Supplementary Figure 1. Study flow chart of participant inclusion and exclusion** 9](#_Toc214817460)

**Supplementary Methods 1. Protocols of Aβ PET imaging acquisition**

**1.1 K-ROAD**

In the K-ROAD study, amyloid PET imaging was conducted using one of two tracers: [18F]florbetaben (FBB), or [18F]flutemetamol (FMM). All scans were acquired in 3D mode using Discovery STe PET/CT scanners across participating sites.

For PiB PET, an average dose of 420 MBq was administered, followed by CT-based attenuation correction and a 30-minute static emission scan capturing 35 slices at 4.25 mm thickness. For FBB and FMM, participants received 311.5 MBq or 197.7 MBq, respectively, and underwent 20-minute dynamic scans beginning 90 minutes after tracer injection. These scans encompassed 47 axial slices with a thickness of 3.3 mm.

All PET images were reconstructed using an ordered-subsets expectation maximization (OSEM) algorithm with 4 iterations and 20 subsets. The reconstructed image matrix was 128 × 128 × 48 voxels, with a voxel size of 2 × 2 × 3.27 mm³.

Preprocessing of FBB and FMM data were processed using SPM8 within Matlab 2014b. PET volumes were co-registered to individual structural MRIs and spatially normalized to MNI-152 standard space. Brain regions were segmented using the automated anatomical labeling (AAL) atlas to enable regional analyses.

**1.2 NA-ADNI Cohort**

Amyloid PET imaging in the NA-ADNI cohort was performed using [18F]florbetapir. Participants received an intravenous bolus of approximately 370 MBq (10 mCi) of the tracer, followed by PET acquisition beginning 50 to 70 minutes post-injection. Scanning consisted of four consecutive 5-minute frames in 3D dynamic mode. The acquired frames were realigned, averaged, and resampled to a standardized voxel dimension of 1.5 × 1.5 × 1.5 mm³, then smoothed to achieve an 8 mm³ full width at half-maximum (FWHM) resolution.

Each participant’s structural T1-weighted MRI (MPRAGE) was used for anatomical co-registration and cortical surface segmentation using FreeSurfer. Cortical and reference regions were defined in native space. Further technical details regarding PET acquisition protocols are publicly available via the ADNI resource (<http://www.loni.ucla.edu/ADNI>).

**Supplementary Methods 2. Protocols of plasma collection for the K-ROAD study**

A total of 8 mL of blood was drawn from each participant and laced in tubes containing 0.5 M ethylenediaminetetraacetic acid. The samples were agitated for 5 min and then centrifuged at 1300×g for 10 min, separating the plasma into 5–10 vials, each containing 0.3 mL. The plasma samples were then stored at -75 ºC until they were analyzed. The procedures were conducted in accordance with the guidelines by the National Biobank of the Republic of Korea for human resource collection and registration.

**Supplementary Table 1** Cost-based assessment of pTau217 cutoffs for therapy stratification including Aβ-positive and Aβ-negative participants.

| Cohort | Assay (n) | Strategy | FN (n) | FP (n) | Intermediate (n) | Total Cost  (1,000 USD) |
| --- | --- | --- | --- | --- | --- | --- |
| K-ROAD | UGOT-Simoa  (n=120) | Single | 8 | 11 | – | 394 |
|  |  | Double | 5 | 6 | 24 | 316 |
|  | Lilly-MSD  (n=120) | Single | 16 | 4 | – | 248 |
|  |  | Double | 5 | 6 | 23 | 312 |
| NA-ADNI | Quanterix-Simoa  (n=280) | Single | 8 | 63 | – | 1,954 |
|  |  | Double | 6 | 21 | 109 | 1,114 |
|  | C2N ratio  (n=171) | Single | 17 | 26 | – | 916 |
|  |  | Double | 9 | 11 | 84 | 738 |
|  | Fujirebio-Lumipulse (n=269) | Single | 6 | 64 | – | 1,968 |
|  |  | Double | 6 | 25 | 56 | 1,022 |
|  | Janssen-Simoa  (n=27) | Single | 14 | 31 | – | 1,042 |
|  |  | Double | 6 | 26 | 93 | 1,200 |

Abbreviations: pTau217, plasma phosphorylated tau 217; Aβ+, β-amyloid positive; K-ROAD, Korea–Registries to Overcome Alzheimer’s Disease and Accelerate Dementia; NA-ADNI, North American Alzheimer’s Disease Neuroimaging Initiative; UGOT-Simoa, ALZpath antibody on Simoa platform (University of Gothenburg); Lilly-MSD, customized pTau217 assay on MSD (Lilly Research Labs); Quanterix-Simoa, ALZpath antibody on Simoa platform (Quanterix); C2N ratio, LC–MS/MS–based pTau217/non-pTau217 ratio (C2N Diagnostics); Fujirebio-Lumipulse, pTau217 assay on Lumipulse G platform (Fujirebio); Janssen-Simoa, Janssen-developed pTau217 assay on Simoa platform (Janssen); FN, false negative; FP, false positive; USD, United States dollar.

**Supplementary Table 2** Cost-based assessment of pTau217 cutoffs for therapy stratification in Aβ-positive cognitively impaired participants.

| Cohort | Assay (n) | Strategy | FN (n) | FP (n) | Intermediate (n) | Total Cost  (1,000 USD) |
| --- | --- | --- | --- | --- | --- | --- |
| K-ROAD | UGOT-Simoa  (n=86) | Single | 7 | 3 | – | 146 |
|  |  | Double | 4 | 1 | 20 | 182 |
|  | Lilly-MSD  (n=86) | Single | 14 | 2 | – | 172 |
|  |  | Double | 4 | 3 | 15 | 182 |
| NA-ADNI | Quanterix-Simoa  (n=63) | Single | 2 | 20 | – | 616 |
|  |  | Double | 2 | 8 | 32 | 384 |
|  | C2N ratio  (n=58) | Single | 7 | 11 | – | 386 |
|  |  | Double | 3 | 4 | 28 | 256 |
|  | Fujirebio-Lumipulse (n=59) | Single | 1 | 21 | – | 638 |
|  |  | Double | 1 | 10 | 17 | 376 |
|  | Janssen-Simoa  (n=60) | Single | 6 | 11 | – | 378 |
|  |  | Double | 2 | 9 | 24 | 382 |

Abbreviations: pTau217, plasma phosphorylated tau 217; Aβ+, β-amyloid positive; K-ROAD, Korea–Registries to Overcome Alzheimer’s Disease and Accelerate Dementia; NA-ADNI, North American Alzheimer’s Disease Neuroimaging Initiative; UGOT-Simoa, ALZpath antibody on Simoa platform (University of Gothenburg); Lilly-MSD, customized pTau217 assay on MSD (Lilly Research Labs); Quanterix-Simoa, ALZpath antibody on Simoa platform (Quanterix); C2N ratio, LC–MS/MS–based pTau217/non-pTau217 ratio (C2N Diagnostics); Fujirebio-Lumipulse, pTau217 assay on Lumipulse G platform (Fujirebio); Janssen-Simoa, Janssen-developed pTau217 assay on Simoa platform (Janssen); FN, false negative; FP, false positive; USD, United States dollar.

**(A) K-ROAD cohort**

**(B) NA-ADNI cohort**

Eligible ADCI participants with

tau PET scans (n = 456)

Eligible ADCI participants with

tau PET scans (n=190)

Exclusion criteria met (n=176)

• Plasma p-tau217 not available
• Outliers excluded (mean±3 SD)

Exclusion criteria met (n=70)

• Plasma p-tau217 not available
• Outliers excluded (mean±3 SD)

Final ADNI participants (n=280)

CU/MCI/DAT (n= 172/81/27)

Final Korean cohort (n=120)

CU/MCI/DAT (n= 26/41/53)

Assay-specific quality control

LOD-imputed values excluded

Assay-specific quality control

LOD-imputed values excluded

- **Quanterix-Simoa** (n = 280)
- **C2N ratio** (n = 171)
- **Fujirebio Lumipulse** (n = 269)
- **Janssen-Simoa** (n = 277)
- **UGOT-Simoa** (n = 120)
- **Lilly-MSD** (n = 120)

Amyloid-β- positive participants

Amyloid-β- positive participants

- **Quanterix-Simoa** (n = 136)
- **C2N ratio** (n = 114)
- **Fujirebio Lumipulse** (n = 132)
- **Janssen-Simoa** (n = 133)
- **UGOT-Simoa** (n = 100)
- **Lilly-MSD** (n = 100)

**Supplementary Figure 1. Study flow chart of participant inclusion and exclusion.**

Abbreviations: p-tau217, plasma phosphorylated tau at threonine 217; K-ROAD, Korea–Registries to Overcome Dementia and Accelerate Dementia; NA-ADNI, North American Alzheimer’s Disease Neuroimaging Initiative; ADCI, Alzheimer’s disease–related cognitive impairment; CU, cognitively unimpaired; MCI, mild cognitive impairment; DAT, dementia of Alzheimer’s type; tau PET, tau positron emission tomography; LOD, limit of detection; UGOT-Simoa, ALZpath antibody–based p-tau217 assay on the Simoa platform (University of Gothenburg); Lilly-MSD, customized p-tau217 assay on the Meso Scale Discovery platform (Lilly Research Laboratories); Quanterix-Simoa, ALZpath antibody–based p-tau217 assay on the Simoa platform (Quanterix); C2N ratio, LC–MS/MS–based p-tau217/non-p-tau217 ratio (C2N Diagnostics); Fujirebio-Lumipulse, p-tau217 assay on the Lumipulse G platform (Fujirebio); Janssen-Simoa, Janssen-developed p-tau217 assay on the Simoa platform (Janssen).
